# Naturalistic fMRI Mapping Reveals Superior Temporal Sulcus as the Hub for the Distributed Brain Network for Social Perception

**DOI:** 10.3389/fnhum.2012.00233

**Published:** 2012-08-13

**Authors:** Juha M. Lahnakoski, Enrico Glerean, Juha Salmi, Iiro P. Jääskeläinen, Mikko Sams, Riitta Hari, Lauri Nummenmaa

**Affiliations:** ^1^Brain and Mind Laboratory, Department of Biomedical Engineering and Computational Science, School of Science, Aalto UniversityEspoo, Finland; ^2^Advanced Magnetic Imaging Centre, School of Science, Aalto UniversityEspoo, Finland; ^3^Brain Research Unit, O.V. Lounasmaa Laboratory, School of Science, Aalto UniversityEspoo, Finland; ^4^Turku PET Centre, University of TurkuTurku, Finland

**Keywords:** social brain, posterior STS, face, speech, pain, body, social interaction, goal-oriented action

## Abstract

Despite the abundant data on brain networks processing static social signals, such as pictures of faces, the neural systems supporting social perception in naturalistic conditions are still poorly understood. Here we delineated brain networks subserving social perception under naturalistic conditions in 19 healthy humans who watched, during 3-T functional magnetic resonance imaging (fMRI), a set of 137 short (approximately 16 s each, total 27 min) audiovisual movie clips depicting pre-selected social signals. Two independent raters estimated how well each clip represented eight social features (faces, human bodies, biological motion, goal-oriented actions, emotion, social interaction, pain, and speech) and six filler features (places, objects, rigid motion, people not in social interaction, non-goal-oriented action, and non-human sounds) lacking social content. These ratings were used as predictors in the fMRI analysis. The posterior superior temporal sulcus (STS) responded to all social features but not to any non-social features, and the anterior STS responded to all social features except bodies and biological motion. We also found four partially segregated, extended networks for processing of specific social signals: (1) a fronto-temporal network responding to multiple social categories, (2) a fronto-parietal network preferentially activated to bodies, motion, and pain, (3) a temporo-amygdalar network responding to faces, social interaction, and speech, and (4) a fronto-insular network responding to pain, emotions, social interactions, and speech. Our results highlight the role of the pSTS in processing multiple aspects of social information, as well as the feasibility and efficiency of fMRI mapping under conditions that resemble the complexity of real life.

## Introduction

During recent years, the neural underpinnings of social cognition have captured substantial interest, and several functional neuroimaging studies have strived to elucidate how the human brain parses the social world. Prior studies on brain basis of social cognition have examined, for example, the neural processing of still images of faces (Kanwisher et al., 1997; Rossion et al., 2003; Ishai et al., 2005) or point-light displays of biological motion vs. random or rigid motion (Grézes et al., 2001; Grossman and Blake, 2002). Higher-level aspects of social cognition have been studied, for example, by presenting cartoons and stories that evoke “theory of mind” processes (Gallagher et al., 2000), or geometrical shapes moving in ways that can be interpreted as social and intentional (Castelli et al., 2000).

These studies have highlighted the pivotal role of the superior temporal sulcus (STS) region in processing audiovisual social information (Allison et al., 2000; Hein and Knight, 2008; Nummenmaa and Calder, 2009). The posterior STS (pSTS) has been linked to processing of faces (Haxby et al., 2000; Hoffman and Haxby, 2000), biological motion (Grézes et al., 2001; Grossman and Blake, 2002), and theory of mind (David et al., 2008), whereas the anterior STS has been shown to participate in for example coding of gaze direction in observed faces (Calder et al., 2007). Additionally, the STS region also has an important role in voice processing (Belin et al., 2000).

Nevertheless, social information processing is distributed widely in the brain, involving – in addition to the STS – other specialized brain regions and networks (see reviews in Allison et al., 2000; Frith and Frith, 2003; Olsson and Ochsner, 2008; Hari and Kujala, 2009; Nummenmaa and Calder, 2009). For example, the fusiform gyri have been linked to processing of invariant aspects of faces (Kanwisher et al., 1997; Rossion et al., 2003; Ishai et al., 2005) and of bodies (Peelen and Downing, 2007). Additionally, the inferior occipital, temporal, and parietal areas participate in face perception (Haxby et al., 2000) and the temporo-occipital extrastriate body area subserves processing of bodies (Peelen and Downing, 2007). Moreover, the amygdala is centrally involved in processing of emotional signals, such as facial expressions (Breiter et al., 1996; Morris et al., 1996), and the putative “mirror-neuron system” of parietal and premotor cortices (Rizzolatti and Craighero, 2004) has been linked to the understanding of other people’s goal-directed actions.

Until now, however, the majority of neuroimaging studies on social cognition have focused on either a single social cognitive function at a time, or on comparison of two opposing social categories (e.g., perception of faces vs. bodies). While these studies have significantly improved our understanding of the neural basis of social cognition, the obvious drawback of this type of approach is the scattered nature of the findings. Moreover, testing only a single feature or a contrast between two features in a given experiment may overlook other possible explanations for the observed activations. Even more importantly, presentation of impoverished, static social stimuli in clearly defined block designs often lack ecological validity.

Recently many perceptual brain functions have been successfully studied in rich stimulus environments approaching the complexity of real life (e.g., Bartels and Zeki, 2004; Malinen et al., 2007; Ylipaavalniemi et al., 2009; Alluri et al., 2012; Lahnakoski et al., 2012), indicating the feasibility of such experimental approaches, despite the obvious challenges in signal analysis due to the complexity of the recorded signals. These studies show that several aspects of brain function, such as face, body, and language perception (Bartels and Zeki, 2004), can be investigated in more naturalistic experimental conditions than have typically been employed in neuroimaging studies.

In the present study we developed an functional magnetic resonance imaging (fMRI) experiment design, in which careful pre-selection and subjective rating of brief movie stimuli provided strict experimental control over the complex stimulation. We included multiple social features in a single experiment to test multiple alternative hypotheses in the same stimulus conditions so that the analyses were not artificially limited to certain sub-functions of complex social processing.

Using this naturalistic audiovisual fMRI mapping approach, our goal was to characterize brain networks processing different features of social stimuli. We generated a large database of short movie clips, each depicting prominently one or more of eight social features (faces, human bodies, biological motion, intentional action, emotion, social interaction, pain, and speech) and one or more of six non-social features (places, objects, rigid motion, people not in social interaction, non-goal-oriented action, and non-human sounds). Non-human sounds included all other sounds except human voice, such as animals, music, and environmental sounds.

The moment-to-moment prominence of each feature in the film clips was rated and used to predict the fMRI signals, resulting in an efficient mapping of brain networks subserving perception of each of the eight social features.

## Materials and Methods

### Subjects

Nineteen healthy subjects (21–34 years, mean 28; 16 males and 3 females; 14 right-handed, 5 left-handed) participated in the study. Individuals with diagnosed neurological or psychiatric disorders or current medication affecting the central nervous system were excluded. Data from one additional subject were lost due to technical problems. All subjects provided informed consent as a part of the protocol approved by the ethics committee of the Hospital District of Helsinki and Uusimaa. The study was carried out in accordance with the guidelines of the declaration of Helsinki.

### Stimuli

**Figure 1:**
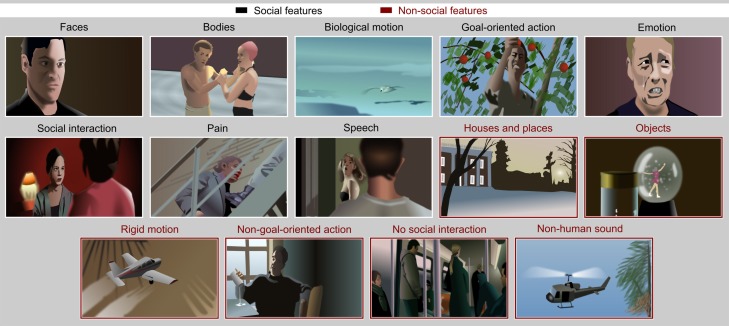
**Examples of movie scenes representing each feature included in the study**. The social and non-social features are indicated with white and red frames, respectively. All examples are drawings created for illustration purposes only; the actual movies involved human actors and natural scenery.

Participants watched short excerpts (mean ± SD duration 16.6 ± 2.7 s, range 10.3–27.3 s) of feature movies. Figure 1 gives schematic examples of the eight social features (faces, bodies, biological motion, goal-oriented action, emotion, social interaction, pain, and speech) and of the six features lacking social content (houses, objects, rigid motion, non-goal-oriented action, humans not participating in social interaction, and non-human sounds) depicted in the movie clips. To allow separation of human actions and other forms of biological motion, clips depicting human movements were complemented with seven clips showing animal and other natural motion. The non-human sounds included animal sounds in seven clips and one clip included sounds of the animal or animals visually depicted in the clip. Seventy five clips contained background music and one clip depicted a band playing. Other non-human sounds were mechanical noises and environmental sounds.

The stimulus set had been validated in a pilot study, for which we had first extracted 192 clips from the movies clearly depicting the targeted social and non-social features. One clip was allowed to depict more than one feature, for example both faces and houses, and 17–20 clips per feature were selected. Five individuals then watched the clips on a computer screen and gave single ratings for the prominence of each social and non-social feature for each clip using a scale from 1 (not present at all) to 5 (present very clearly). On the basis of these ratings, we selected the final 137 clips to be used in the fMRI experiment. For each feature, we selected clips that received the highest ratings for the *a priori* target feature while being as independent as possible from the other features. For example, bodies could be prominently visible in a subset of the scenes depicting social interaction while other clips displaying social interaction contained little or no visible bodies (e.g., close-up pictures of interacting faces), thus making the correlation between these features low.

The selected clips were subsequently rated in detail by two independent persons who gave continuous ratings of the social and non-social features depicted in the movie clips using a web-based rating tool (see Appendix for the instructions for the ratings). While viewing each movie, the raters moved with a mouse a small cursor up and down on the edge of the screen to continuously indicate how prominently the currently rated feature was present in the film. Each trial started with a text describing the feature to-be-rated from the movie clip. After the rater clicked the mouse button, a still image of the first frame of the upcoming movie clip was presented for 5 s allowing the raters to select their initial rating for the first frame before the video started. Different features were rated on separate runs for each clip in random order. The ratings were sampled at 5 Hz. The time series of the behavioral ratings for each feature were averaged over the two raters and organized by concatenating them in the order in which the corresponding videos were presented during the fMRI experiment (see below). Next, these time courses, down-sampled to match the repetition time (2112 ms) of the fMRI data acquisition, were used as regressors in the fMRI analysis.

### Experimental design for fMRI

In the scanner, the participants watched the clips in a fixed pseudorandom order that contained no gaps between the clips. The stimuli were delivered using Presentation software (Neurobehavioral Systems Inc., Albany, CA, USA), and they were back-projected on a semitransparent screen using a 3-micromirror data projector (Christie X3, Christie Digital Systems Ltd., Mönchengladbach, Germany), and from there via a mirror to the subject. The viewing distance was 34 cm, and the width of the projected image was 19.7 cm. The subjects were instructed to view the clips similarly as if they would watch movies from TV or at cinema. The audio track of the movie was played to the subjects with an UNIDES ADU2a audio system (Unides Design, Helsinki, Finland) via plastic tubes through porous EAR-tip (Etymotic Research, ER3, IL, USA) earplugs. Sound intensity was adjusted to be loud enough to be heard over the scanner noise and was individually fine-tuned between ±2 dB to a comfortable level. Total scanning time was 27 min 6 s.

### fMRI acquisition and analysis

MR imaging was carried out with 3.0-T GE Signa VH/i MRI scanner with HDxt upgrade (GE Healthcare Ltd., Chalfont St Giles, UK) using a 16-channel receiving head coil (MR Instruments Inc., MN, USA). Whole-brain data were acquired with T_2⋆-weighted echo-planar imaging (EPI), sensitive to the blood oxygenation dependent (BOLD) contrast (36 axial slices acquired in interleaved ascending order, no gaps, 4-mm slice thickness, TR = 2112 ms, TE = 30 ms, in-plane resolution 3.75 mm × 3.75 mm, acquisition matrix = 64 × 64 voxels, flip angle = 75°). Each dataset consisted of 770 functional volumes, and the first two volumes were discarded to allow for equilibration effects. Anatomical images with 1-mm isotropic voxels were acquired with a *T*_1_-weighted spoiled gradient echo (SPGR) sequence with ASSET parallel imaging (182 axial slices, no gaps, TR = 10 ms, TE = 1.9 ms, acquisition matrix = 256 × 256, flip angle = 15°).

Functional data were preprocessed with FSL (FMRIB’s Software Library; Smith et al., 2004; Woolrich et al., 2009). First, the images were realigned to middle scan by rigid-body transformations using Motion Correction using FMRIB’s Linear Image Registration tool (MCFLIRT; Jenkinson et al., 2002). Subsequently, bias field was removed from the anatomical images using FMRIB’s Automated Segmentation Tool (FAST; Zhang et al., 2001), and non-brain matter was removed from both anatomical and functional images using Brain Extraction Tool (BET; Smith, 2002). Values for intensity threshold and threshold gradient in BET were searched manually by changing the parameters and visually inspecting each brain extracted volume until the results were optimal. Functional datasets were first co-registered to the subject’s brain extracted *T*_1_-weighted image which was then registered to the MNI152 standard space template with 2-mm resolution. Both co-registration steps were performed using FMRIB’s Linear Image Registration tool (FLIRT; Jenkinson et al., 2002) using nine degrees of freedom (translation, rotation, and scaling).

Functional data were smoothed using a Gaussian kernel with full-width-at-half-maximum (FWHM) value of 6.0 mm. High-pass temporal filtering was applied using Gaussian-weighted least-squares straight line fitting, with standard deviation of 50 s, with the first two volumes of each dataset discarded (a fixation cross was presented during these volumes). Functional images were co-registered manually using Nudge of the FSL suite to improve the automatic co-registration process. This manual adjustment was based on visually identified anatomical landmarks (corpus callosum, cerebrum-cerebellum border, outline of the inferior part of the temporal poles, and the curvature of the cerebral cortex at the top of the head).

### Analysis of regional effects

Data were analyzed with SPM8 software.^1^ A random-effects model was implemented using a two-stage process (first and second-level). For each participant, we used general linear model (GLM) to assess regional effects of the eight social and six non-social features on the BOLD signal. We performed the first-level analysis both without and with orthogonalization of the regressors. We first analyzed the data using single regressors that represented the moment-to-moment intensities of each of the 14 features with no orthogonalization. This analysis served to identify brain regions responding to the social and non-social stimulus features. In the second approach, we orthogonalized each regressor with respect to all other regressors and performed the analysis again to reveal which brain areas were associated with a particular stimulus feature independently of all other features. While this approach is appealing in traditional experiments, the complexity and non-linear dependencies of the parametric models in the current study may lead to spurious results, consequently hampering the interpretation of the orthogonalized regressors. We therefore compared the results obtained by using both the original and orthogonalized regressors in the GLM.

Low-frequency signal drifts were removed by high-pass filtering (cutoff 128 s), and thereafter AR(1) modeling of temporal autocorrelations was applied. For both analysis strategies, individual contrast images were generated for main effects of all social and non-social features. The results of the first-level analyses were subjected to second-level analyses in MATLAB using one-sample *t*-test. Effects of each feature were compared with the null hypothesis of no activation. Additionally, the main effects of social vs. non-social features were compared over subjects to reveal which brain areas showed significant differences between social vs. non-social scenes by comparing the fits of each contrast in a paired-samples *t*-test. Statistical threshold was set at *p* < 0.05 FDR corrected over all brain voxels.

To estimate the relative response amplitudes to each feature, we explored the beta weights of the GLM in brain areas showing prominent overlap of responses to several social features. Mean beta weights were calculated in a spherical volume (radius 3 mm) around the voxels showing maximal overlap of sensitivity to multiple social features. In simple block designs, the beta weights are directly proportional to response amplitude minus baseline activity. Here, due to the complex model, the relation between signal amplitude and beta weight is not equally straightforward, but the beta weights give estimates of the best coefficients for fitting the observed activity with the model of each feature separately.

Finally, to illustrate the similarities of the activity profiles of different brain areas involved in processing of social stimuli we calculated functional connectivity between the regions of interest described above. We extracted the mean BOLD time series of spherical volumes at each region (radius 3 mm) and calculated for each subject the correlation coefficients of the time series with the other regions of interest. Fisher *Z*-transform was applied to the correlation coefficients, mean correlation across subjects was computed, and finally the mean value was inverse transformed. Statistical significance of the mean functional connectivity was assessed by permutation testing using one million permutations. Time courses for regions were calculated as the mean activity within spheres with 6 mm radius to increase signal to noise ratio and obtain a more conservative correlation threshold. Permutations were performed by randomly selecting a seed region for each subject. The seed time course was randomly circularly shifted by at least 10 samples and its correlation with the time courses of all other regions of interest was calculated. Because any correlations in the permutation distribution could only be due to false positives, we calculated the absolute value of the correlation coefficients in the permutation distribution and selected the maximum observed value (|*r*| ≈ 0.435) as the threshold of statistically significant correlation (the threshold for 3-mm radius spheres was |*r*| ≈ 0.402). The community structure of the functional network between the regions of interest was studied using Infomap algorithm^2^ (Rosvall and Bergstrom, 2007, 2008). The algorithm employs random walks as a proxy for the probability of information flow within the network, and divides the main network into sub-networks by compressing the description length of information flow, producing communities of strongly connected areas with few links acting as bridges between the modules. The algorithm was run 100 times with random initializations, and the community structure with the lowest description length was selected. Same community structure was found in 90% of the repetitions. Ten percent of repetitions converged to another, sub-optimal description where fusiform gyri and MT of both hemispheres were classified into the fronto-parietal network while the other module boundaries remained unchanged. Although community structure depends on the correlation threshold between the nodes, in the current case the community structure was otherwise identical with both correlation thresholds, but temporo-frontal and temporo-amygdalar areas were merged into one community when the lower threshold was used.

## Results

**Figure 2:**
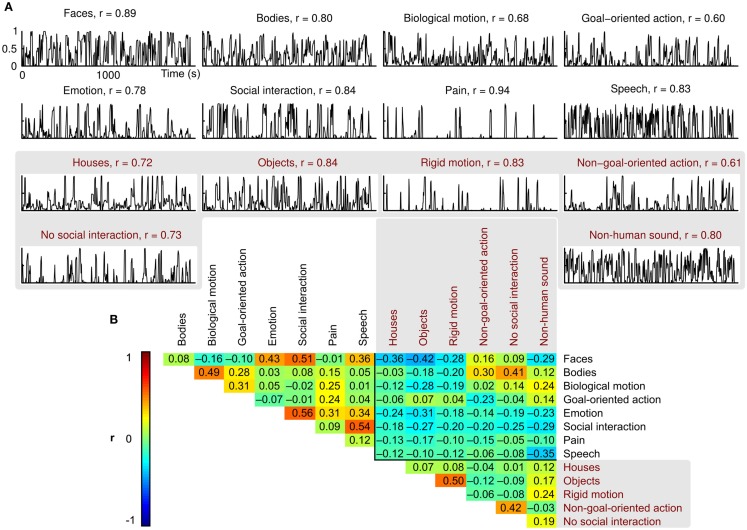
**Behavioral ratings and between-features correlations**. **(A)** Normalized mean ratings for each feature, and the correlation coefficient (*r*) between the two raters. **(B)** Correlation matrix showing the pairwise correlations between the mean ratings of each feature. Correlation coefficients are indicated by both color coding and exact values in the corresponding cells.

Scores of the two independent raters were consistent (mean *r* = 0.78, Figure 2A), and the features were relatively independent of each other (mean between-features |*r*| = 0.18, Figure 2B; green and yellow colors in the correlation matrix). However, relatively high correlations (*r* ≈ 0.5; red) between some feature pairs, such as faces and social interactions or objects and rigid motions, were unavoidable.

**Figure 3:**
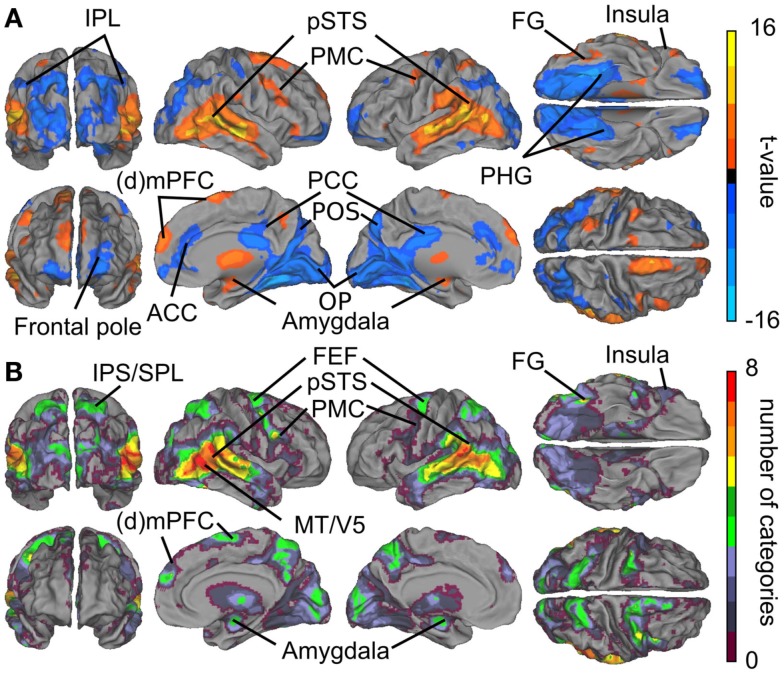
**Brain areas subserving audiovisual social perception**. **(A)** Contrasts between all social vs. all non-social features. Warm colors (orange–yellow) indicate areas that responded more to social than non-social features, and cold colors (blue) indicate areas that reacted more to scenes containing non-social than social features. **(B)** Cumulative activation maps showing how many of the eight social features were associated with statistically significant activity (*p* < 0.05 FDR corrected) in each brain area using non-orthogonalized regressors. Abbreviations: ACC, anterior cingulate cortex; (d)mPFC, (dorso)medial prefrontal cortex; FEF, frontal eye field; FG, fusiform gyrus; IPL, inferior parietal lobule; IPS, intraparietal sulcus; MT/V5, middle temporal visual area; OP, occipital pole. PCC, posterior cingulate cortex; PHG, parahippocampal gyrus; PMC, premotor cortex; POS, parieto-occipital sulcus; pSTS, posterior superior temporal sulcus; SPL, superior parietal lobule.

Figure 3A summarizes the results of the GLM analyses. Contrasts between all social vs. all non-social features revealed that particularly the posterior temporal areas responded more strongly (warm colors) to social than non-social signals. Significantly higher activity to social than non-social features was also observed in the lateral fusiform gyrus (FG), ventral premotor cortex, medial frontal regions, amygdala, and the thalamus. Clear activity was observed bilaterally in the thalamus, with peak activity in the right pulvinar (thalamic activity is occluded in the figure). Conversely, non-social rather than social features elicited stronger activity (cold colors in Figure 3) in anterior cingulate cortex (ACC) and posterior cingulate cortex (PCC), parahippocampal gyri (PHG), and occipital- and parieto-occipital cortices, lateral frontal pole, and posterior aspects of the inferior parietal lobule (IPL).

Figure 3B shows cumulative population maps of feature sensitivity for brain areas significantly activated by each social feature: the warmer the color, the larger number of features activated the region. All eight social features elicited reliable responses in the pSTS region and in a small cluster in the right FG. As is indicated by greenish colors, half of the social features also activated the premotor cortex particularly in the right hemisphere, the frontal eye fields (FEFs) and intraparietal sulcus parts of the dorsal attention network, amygdala, and parts of the dorsomedial prefrontal cortex (dmPFC).

**Figure 4:**
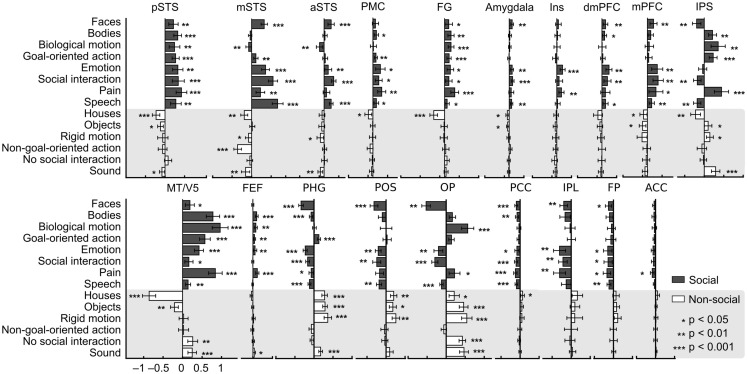
**Beta weights of each feature in the GLM analysis in selected regions**. Weights are based on the non-orthogonalized regressors and each feature was analyzed separately. Social features are plotted with gray bars and non-social features in white bars on gray background. Error bars correspond to the 95% confidence interval of the mean beta weight across subjects. Asterisks indicate features that correlated statistically significantly with the activity in the regions of interest. These data, here plotted for visualization only, were not subjected to secondary statistical analysis. Abbreviations as in Figure 3, in addition aIns, anterior insula.

To illustrate which social features resulted in overlapping activations shown in Figure 3, we extracted the GLM parameter estimates for each regressor in these areas. Figure 4 depicts the mean (+95% confidence interval) beta weights for each feature in regions of interest (for coordinates, see Table 1) that either (i) were more active in non-social than social scenes or (ii) showed locally maximal overlap of sensitivity to social features. Statistical significance of each feature in the regions of interest is based on the original contrasts depicted in Figure 3. We present results only for the hemisphere which showed the stronger cluster in Figure 3 although the results were highly similar in both hemispheres. Values are given separately for the posterior, medial, and anterior parts of the STS (pSTS, mSTS, and aSTS, respectively).

**Table 1:** **MNI coordinates of regions of interest whose beta weights were extracted for the quantification shown in Figure 4**.

Area	Hemisphere	*x*	*y*	*z*
**AREAS RELATED TO SOCIAL FEATURES**				
Posterior STS	Left	−58	−42	12
Posterior STS	Right	58	−44	14
Middle STS	Left	−62	−32	6
Middle STS	Right	60	−22	−2
Anterior STS	Left	−56	−4	−16
Anterior STS	Right	54	4	−28
MT/V5	Left	−54	−62	6
MT/V5	Right	50	−66	−2
Fusiform gyrus	Left	−46	−50	−20
Fusiform gyrus	Right	42	−50	−20
Amygdala	Left	−24	−10	−16
Amygdala	Right	24	−8	−18
Intraparietal sulcus	Left	−26	−52	56
Intraparietal sulcus	Right	28	−56	56
Frontal eye field	Left	−20	−6	62
Frontal eye field	Right	20	−4	62
Premotor cortex	Left	−50	4	32
Premotor cortex	Right	40	16	26
Medial PFC	Left	−4	56	26
Medial PFC	Right	4	54	26
Dorsomedial PFC	Left	−4	10	66
Dorsomedial PFC	Right	14	28	58
Anterior insula	Left	−42	30	4
Anterior insula	Right	44	26	−4
**AREAS RELATED TO NON-SOCIAL FEATURES**				
Anterior cingulate cortex	Right	10	40	8
Posterior cingulate cortex	Left	−6	−26	28
Inferior parietal lobule	Left	−50	−58	44
Frontal pole	Left	−26	58	8
Parahippocampal gyrus	Right	26	−48	−12
Parieto-occipital sulcus	Left	−12	−72	38
Occipital pole	Left	−14	−90	0

*Listed regions showed either (i) prominent overlap of sensitivity to social features or (ii) were activated more by non-social vs. social features*.

Only pSTS shows statistically significant activity and relatively high positive weights to all social features and negative or non-significant weights for all non-social features. FG activity also correlated with all social features and with none of the non-social features, but the parameter estimates for social features were lower and more variable than in the pSTS. Bodies, biological motion, goal-oriented action, emotions, and pain were associated with strong MT/V5 responses, whereas faces, emotions, social interaction, and speech received high weights in the mSTS. In the IPS, highest weights were observed for bodies, biological motion, goal-directed action, and pain but – interestingly – faces and social interaction resulted in negative weights. A very similar response pattern but with lower amplitudes was seen in the precentral sulcus in or in the vicinity of the FEF, the other main node of the dorsal attention network. In the frontal areas (PMC, mPFC, and dmPFC), the highest weights were observed for emotions and social interactions. Pain additionally received positive weights in the PMC and mPFC. Anterior insula (aIns) was sensitive only to pain and emotions. Amygdala received low positive weights for faces, emotions, social interaction, and speech.

Figure 4 further indicates that among the areas showing preference to non-social vs. social categories, the parahippocampal gyrus, parieto-occipital sulcus (POS) region, and occipital pole (OP) are the only areas that significantly correlated with, and received high weights for non-social features. All social features received negative or zero weights in the POS region. Goal-oriented action was the only social feature that was significantly positively correlated with activity in the PHG. Early visual areas in the OP received highly variable weights for different features, but mean weights were higher for non-social vs. social features. Other areas showing significantly higher activity during non-social vs. social features showed more significant negative correlations with social features than positive correlations with non-social features.

**Figure 5:**
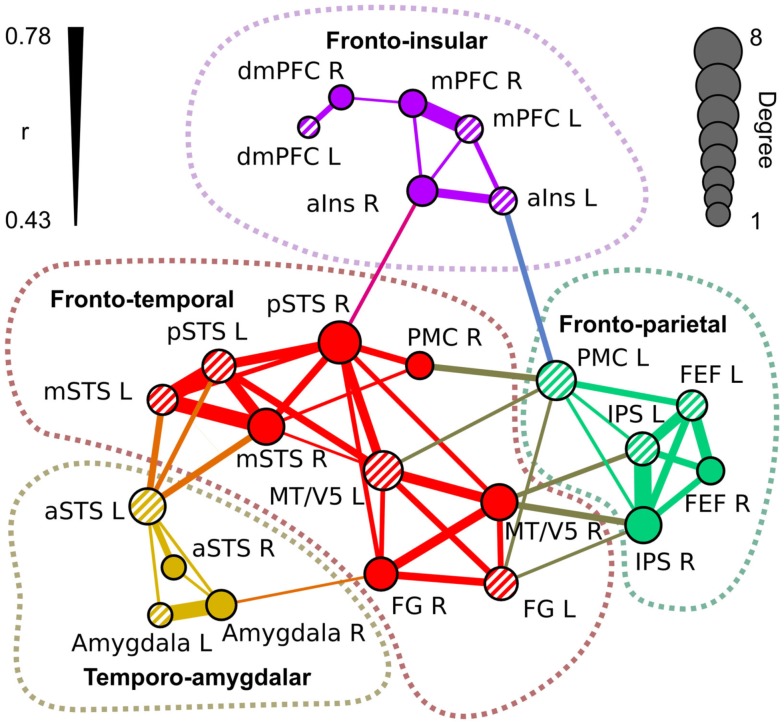
**Functional network structure of the regions of interest in Table 1**. The links indicate statistically significant (*p* < 10^–6^ in permutation distribution) mean functional connectivity (*r*) over subjects between the seed regions. Thickness of the link indicates the correlation coefficient between the areas. The diameter of the nodes indicates the number of links connected to it (degree). Left-hemisphere nodes are indicated by striped colors.

Figure 5 visualizes the functional network structure for regions activated by social categories in Table 1. This network analysis illustrates across-regions similarities in the sensitivity profiles even though a portion of the observed functional connectivity can be explained by similarities in non-stimulus-related hemodynamic activity. Functional connectivity analysis revealed four separate networks: (1) a fronto-temporal network (red nodes and connections) included pSTS, mSTS, MT/V5, FG, and right PMC, (2) a fronto-parietal network (green) comprised IPS, FEF, and left PMC, (3) a temporo-amygdalar network (yellow) included amygdalae and aSTS bilaterally, and (4) a fronto-insular network (purple) comprised mPFC, dmPFC, and aIns. The regional beta weights (Figure 4) were more similar in functionally connected than in non-connected regions.

The fronto-temporal network is widely connected with the fronto-parietal network through MT/V5, PMC, and FG. The temporo-amygdalar network has strong connectivity from left aSTS to bilateral mSTS and left pSTS, and right amygdala is functionally connected to right FG. The fronto-insular network is relatively weakly connected to other regions, with significant correlations only between right insula and right pSTS, and between left insula and left PMC. The fronto-temporal network is located in a key position to integrate all the parts of the network. Only the link between left insula and left PMC connects the fronto-insular and fronto-parietal networks directly.

The nodes of the fronto-temporal network, depicted in Figure 5, showed a rather varied response pattern to different features. While most of the nodes (pSTS, mSTS, and PMC) were responsive to pain, emotions, and social interaction, regional variability in responses to other features was large. The nodes of the fronto-parietal network responded to bodies, biological motion, goal-directed action, and pain. MT/V5 of the fronto-temporal network and FEF of the fronto-parietal network also responded to emotions. The temporo-amygdalar network responded to faces, social interaction, and speech. The fronto-insular network responded consistently to emotions and in a right-lateralized manner to pain. Additionally, dmPFC and mPFC responded to social interaction and speech.

**Figure 6:**
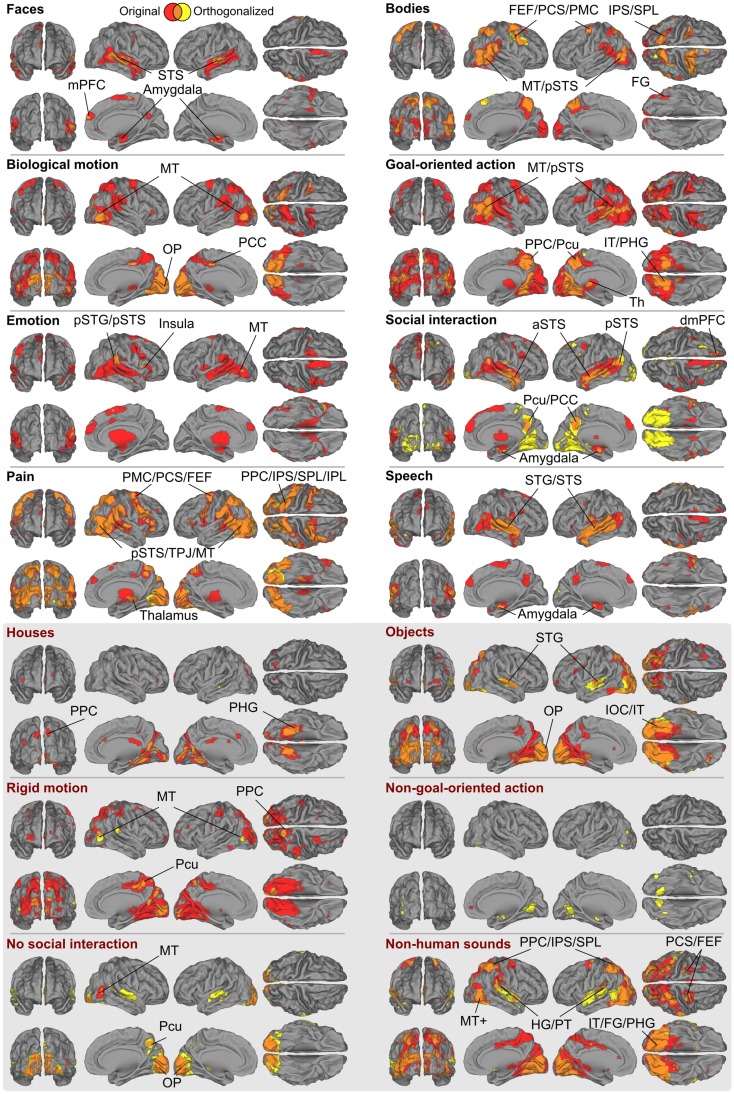
**Brain areas subserving different aspects of social perception**. Red denotes areas whose responses were correlated only with the original ratings, yellow denotes areas that were correlated only with the orthogonalized ratings; overlap is shown in orange. The labeled locations correspond to the areas where the two results overlapped. Abbreviations as in Figure 3. In addition: amy, amygdala; FG, fusiform gyrus; PPC, posterior parietal cortex; SPL, superior parietal lobule; Pcu, precuneus; OP, occipital pole; IT, inferior temporal lobe; IOC, inferior occipital cortex.

Figure 6 shows the full GLM results for the orthogonalized and non-orthogonalized regressors, and their overlap regions. The orthogonalized regressors were applied to unravel areas responding to each feature independently from the other features. Even after orthogonalization, all eight social features still significantly activated areas in or near the pSTS region.

With orthogonalized regressors, faces activated the pSTS/mSTS, and small clusters in the amygdala, and mPFC. Bodies activated the pSTS/MT region, IPS, PMC, dMPFC, and a small cluster in the FG, with more widespread activations in the right hemisphere. Biological motion activated the MT/V5 as well as visual regions in the medial occipital lobe. Goal-oriented actions activated the pSTS region, posterior parietal cortex/precuneus, POS, and inferior temporal visual areas, whereas emotions activated relatively small areas of the right temporoparietal junction and right aIns and the MT/V5 region in the left hemisphere. Activity related to social interactions was spread along the STS, amygdala, precuneus, and inferior temporal visual areas. Pain activated areas very similar to those activated by bodies, but additional activations were observed in the aIns and inferior frontal gyrus. Finally, speech activated widespread temporal-lobe areas, including the STS and the superior and middle temporal gyri, with additional activations in the amygdala.

In general, the brain regions activated in the GLM analysis were more widespread for non-orthogonalized than orthogonalized features, as is expected on the basis of their inter-feature correlations. However, the areas obtained in both analyses typically overlapped (see orange areas in Figure 6) with prominent differences only for three features. Interestingly, the inferotemporo-occipital surface was widely activated by the orthogonalized social interaction stimuli, covering both fusiform and lingual gyri. Other clear activations were found in the superior temporal gyri of both hemispheres for no social interaction and non-human sounds features.

**Figure 7:**
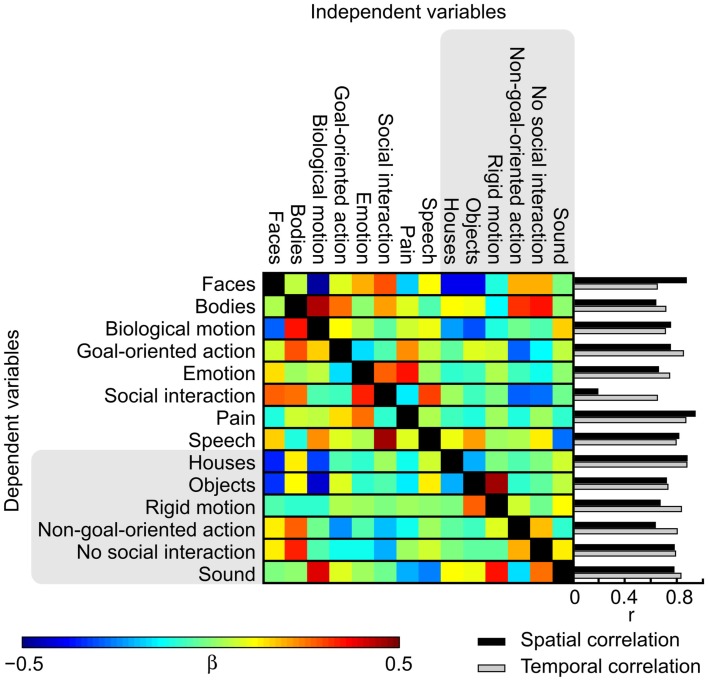
**Weights used in the orthogonalization of the features and spatial and temporal correlations between the original and orthogonalized analyses**. Colors correspond to the beta weights in the fitting process from which the orthogonal residuals were acquired. Bar graphs show the spatial correlation of the *t*-maps of the original and orthogonalized GLMs (black) and temporal correlations between the original and orthogonalized features (gray).

To address the causes underlying the differences between the activation patterns in the orthogonalized vs. original GLM, we studied the effects of orthogonalization in detail. Figure 7 shows the weights used for orthogonalizing the regressors in a linear model and the spatial correlations of the *t*-maps and the temporal correlations of the regressors. While the activation maps obtained with orthogonalized and non-orthogonalized regressors show marked differences for a number of modeled features in the thresholded results, the average spatial correlations (calculated over voxels within the brain) between the *t*-maps resulting from the analysis with orthogonalized and original regressors are high [*r* = (0.63, 0.94)] for all other features except social interaction (*r* = 0.19) that is most critically dependent on the other, lower-level social features. In the beta weight matrix, positive (negative) weights indicate features that were subtracted from (added to) the original time course to create the orthogonal residual. Contribution of bodies, emotions, and speech is essentially removed from social interactions while non-goal-oriented action, absence of social interaction, and pain are added. These features together explained more of the original signal than the orthogonalized regressor did. Accordingly, while the annotated features were not linearly interdependent an obvious dependence between the different features still remained. Consequently, the social interaction feature is difficult to interpret when the additional features are orthogonalized out. Obviously scrutiny is needed in investigating the orthogonalized variables and in comparing the results when multiple complex and dynamic variables are used as predictors.

**Figure 8:**
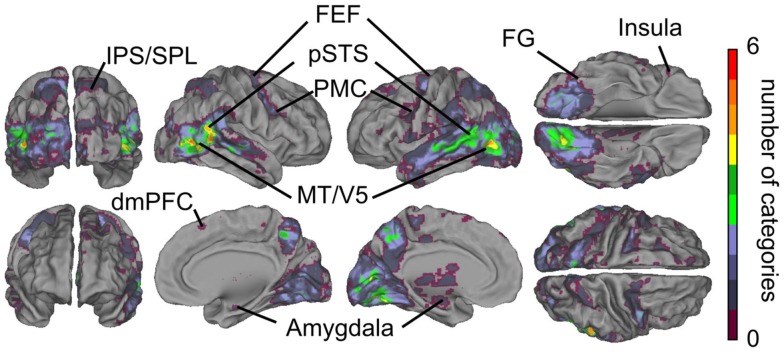
**Overlap of areas reacting to orthogonalized social features**. Cumulative activation maps showing how many of the eight social features were associated with statistically significant activity (*p* < 0.05 FDR corrected) in each brain area using orthogonalized regressors. Abbreviations as in Figure 3.

Figure 8 shows the overlap of social categories using the orthogonalized regressors. Compared with the overlap results obtained with the original regressors (see Figure 3B), the activity foci were now more clearly separated. However, regions with the highest overlap were located in similar regions as in Figure 3B, but only 5–6 orthogonalized features overlapped in the pSTS and MT/V5 regions, compared with 8 in the original analysis.

## Discussion

Using carefully selected and rated film clip stimuli we were able to demonstrate the pivotal role of pSTS in processing of audiovisual social features in a rich, dynamic stimulus stream. STS responded reliably to all eight tested social features, whereas other brain areas had more narrowly tuned response profiles toward specific social features, such as emotions or human bodies. Prior research on different aspects of social perception of isolated social categories has implicated pSTS involvement in several social tasks (for reviews, see Allison et al., 2000; Blakemore, 2008; Hein and Knight, 2008; Hari and Kujala, 2009; Nummenmaa and Calder, 2009). However, our study is the first to show how the pSTS participates in processing of several different social features, all tested in a single “social-localizer” type experiment. Importantly, pSTS was the only brain region showing high selectivity to social in contrast to non-social stimulus features.

### STS as a hub of social processing

Allison et al. (2000) argued that the STS has a general role in social perception, potentially integrating “what” and “where” information from others’ actions. Hein and Knight (2008) took the view that the function of STS depends largely upon the co-activations of connected areas. On the contrary, Haxby et al. (2000) proposed that the pSTS encodes and tracks particularly quickly changing social features, such as facial expressions, whereas recently Nummenmaa and Calder (2009) proposed that the pSTS would be tuned even more narrowly to intentionality of agents’ actions. The present data suggest that the pSTS plays a very general role in social perception, as (i) it responded significantly to all tested social features even after orthogonalization, (ii) it responded with equal magnitude to all the tested social features, and (iii) it showed the strongest preference to social in contrast to non-social stimulus features.

Our results thus suggest that the pSTS is involved in processing multiple social features. But what criteria could the pSTS use for categorizing sensory inputs as “social” vs. “non-social”? One possibility is that the pSTS indeed serves as a “hub” that integrates social information processed in functionally connected sub-systems (see, e.g., Hein and Knight, 2008) rather than being specifically tuned to numerous social features. Our functional connectivity analysis indeed suggests that the pSTS region is functionally tightly coupled with the other brain circuitries that process social information with more narrow tuning.

### Brain networks for social perception

We were able to delineate four partly interconnected networks involved in processing of distinct aspects of social information. First, the fronto-temporal network comprised areas heavily connected with the pSTS, that is the right PMC, and the MT/V5 and FG regions of both hemispheres. This network also appeared to be the key network mediating connectivity between other putative social brain networks. The temporo-amygdalar network, comprising regions in the anterior STS and amygdala, was sensitive to social communication, including speech, as well as to communication of emotions through facial expressions and/or body language. This reactivity is consistent with prior studies showing STS’s sensitivity to speech and voice (Belin et al., 2000; Scott and Johnsrude, 2003; Saur et al., 2008) and the observed sensitivity of amygdala to emotional facial and bodily cues (Breiter et al., 1996; Morris et al., 1996; de Gelder et al., 2010). The core of the fronto-parietal network is the dorsal attention network, including the intraparietal sulci and FEFs of both hemispheres, further connected to the left premotor cortex. This network was most closely connected to embodied aspects of social cognition, such as pain, biological motion, and goal-directed actions. Finally, the fronto-insular network, comprising the aIns and the medial prefrontal cortex of both hemispheres, reacted to emotions and pain, in line with prior studies (Apkarian et al., 2005; Leknes and Tracey, 2008). These networks may of course comprise other nodes that were not included in the present analysis where our main goal was to group together functionally similar brain areas sensitive to a large variety of social features.

Slightly surprisingly, relatively little activity was observed in the medial parts of the prefrontal cortex whose role in social cognition is well established (Amodio and Frith, 2006). However, the medial PFC is typically engaged in internal processing (Lieberman, 2007), such as mentalizing of other persons’ intentions. While the subjects in the current study may have spontaneously recruited their theory of mind skills to make sense of the presented scenes, it is likely that without an explicit task they mostly employed automatic, externally focused perceptual strategies that do not so strongly involve the medial PFC.

### fMRI mapping with naturalistic stimuli as a tool to simultaneously study multiple aspects of social cognition

Our results highlight the feasibility of naturalistic audiovisual stimulation as an efficient way to study multiple aspects of social perception with a “single shot.” During a scanning session of less than half an hour we could map brain processing of eight social features, which would have required multiple lengthy experiments – or even meta-analyses – with block designs and static stimuli. While strictly controlled experimental designs have immensely extended our knowledge of the brain basis of social cognition, the meta-analytic consolidation of information across studies with different types of experimental designs depends on the assumptions made to merge the results of different studies. Consequently, direct tests of multiple facets of social cognition in a single experiment significantly complement meta-analyses in forming a cohesive picture of the brain basis of social cognition, now studied in the same subjects.

To understand the underpinning of social cognition in real life we have to study effects of natural social environment on brain function. We also need to assess whether the results of simplified experimental paradigms generalize to naturalistic situations, and what additional brain mechanisms are needed to integrate the multiple overlapping social signals into a unified percept. Recent work in cognitive neuroscience has taken the first steps toward analyzing how humans parse the dynamic, naturalistic environment. For example, free viewing of movies and video clips elicits reliable activity across subjects in several brain areas (Hasson et al., 2004; Malinen et al., 2007; Jääskeläinen et al., 2008), and post-experiment annotations of specific features of interest from movie stimuli can be used to map brain areas sensitive to the selected stimulus features (Bartels and Zeki, 2004; Ylipaavalniemi et al., 2009; Lahnakoski et al., 2012).

As completing the large number of ratings (1918 in total) took almost three full working days per rater, we gathered complete ratings only from two persons. The to-be-rated categories were unambiguous and clearly defined sensory events, resulting in high inter-rater agreement (mean *r* = 0.78, see Figure 2). Moreover, approximately three quarters of the clips and features were validated by two additional raters, and an additional one quarter by one of the two additional raters. The ratings for this extended subject pool were consistent with the ratings used in this study (*r* > 0.9 for all features).

Although the number of individual contrasts in the current experiment was relatively high, the results we report were not corrected for the number of modeled features because we were interested in the sensitivity of brain areas to social vs. non-social categories rather than to single features. While the most significant activations for each category survived Bonferroni correction by the number of features (14), the overlap between areas sensitive to different social features decreased. However, even with the additional correction, a portion of the right pSTS was still sensitive to all social features.

## Conclusion

We have introduced a novel approach for studying the neural basis of social perception in a highly complex audiovisual stimulus environment. The main advantage of the present approach is that it enables simultaneous assessment of the effects of multiple ecologically valid social stimuli. Testing these alternatives in traditional fMRI designs would obviously lead to prolonged experiments with several independent stimulus categories, whereas our approach enables testing of multiple functional hypotheses simultaneously.

The results, including network and connectivity analyses, suggest that pSTS has a central role in perception of multiple social features, discriminating between social vs. non-social features with very broadly tuned preference; pSTS likely integrates social signals processed by more specialized sub-systems. This novel “social-localizer” approach bridges the gap between classical model-based and more recent model-free analyses of human brain function during social perception.

Future studies should address temporal modulations of the connectivity patterns of the large-scale neuronal networks, for which our current results provide a solid starting point.

## Conflict of Interest Statement

The authors declare that the research was conducted in the absence of any commercial or financial relationships that could be construed as a potential conflict of interest.

## Footnotes

^1^http://www.fil.ion.ucl.ac.uk/spm

^2^http://www.tp.umu.se/∼rosvall/code.html

## Appendix

### Instructions for rating of stimulus features – translated from Finnish

#### General instructions

Rating is done using the mouse. When each movie clip begins you will see the first frame of the clip. In the top part of the screen you will see which feature, for example faces, you are supposed to rate.

By moving the mouse you can move the scroll bar on the right side of the screen to indicate how much you see the rated feature in the first frame (i.e., faces in this example). If the bar is all the way at the bottom it means you do not see the current feature (faces in this case) at all in the image. If the bar is all the way at the top it means the feature (faces in this case) is very prominently present in the image. Move the bar to indicate what you see in the first frame.

When you click on the mouse the movie will begin. After the movie begins you can move the bar at any time during viewing of the movie. Try to follow you perception continuously during the movie and move the bar accordingly. The feature being rated changes from one movie to the next, so be careful and follow the feature you are supposed to rate!

#### Feature specific instructions

##### Faces

How clearly or how many faces are visible in the movie?

##### Houses and places

How clearly or how many houses or different interior/exterior places are visible in the movie?

##### Human bodies

How clearly or how many bodies are visible in the movie?

##### Inanimate objects

How clearly or how many inanimate objects like lamps, chairs, or cars are visible in the movie?

##### Biological motion

How clearly or how much motion of biological beings like humans, animals, and plants, or other natural motion like waves is visible in the movie?

##### Rigid, non-biological motion

How clearly or how much motion of non-biological beings like cars, doors, clock hands, or other mechanical devices is visible in the movie?

##### Goal-oriented action

How clearly or how much goal-oriented action, such as picking apples, opening doors or drawers, combing hair or other such activities is visible in the movie?

##### Non-goal-oriented action

How clearly or how much action which does not have a clear goal, such as sitting idly, scratching matches with no particular reason, or walking with no goal is visible in the movie?

##### Emotions

How clearly or how much emotions or expression of emotions such as emotional facial expressions, crying, laughter, or emotional bodily expressions such as hugging, or emotional speech is visible in the movie?

##### Social interaction

How clearly or how much social interaction between human such as conversation, chatting, or shared activities is visible in the movie?

##### Humans, but no social interaction

How clearly or how many people who are not in social interaction are visible in the movie? They may, for example, be walking side by side or be in the same place but they are not in social interaction with each other.

##### Pain

How many people in pain or how strong pain is visible in the movie.

##### Human speech

How clearly is human speech or other human voice heard in the movie?

##### Non-human sound

How clearly are other sounds than human speech heard in the movie?
